# Evidence from 3-month-old infants shows that a combination of postnatal feeding and exposures *in utero* shape lipid metabolism

**DOI:** 10.1038/s41598-019-50693-0

**Published:** 2019-10-04

**Authors:** Samuel Furse, Stuart G. Snowden, Laurentya Olga, Philippa Prentice, Ken K. Ong, Ieuan A. Hughes, Carlo L. Acerini, David B. Dunger, Albert Koulman

**Affiliations:** 1 Core Metabolomics and Lipidomics Laboratory, Metabolic Research Laboratories, Institute of Metabolic Science, University of Cambridge, Level 4 Pathology, Cambridge Biomedical Campus, Cambridge, CB2 0QQ UK; 2 Department of Paediatrics, University of Cambridge, Box 116, Cambridge Biomedical Campus, Cambridge, CB2 0QQ UK; 3 MRC Epidemiology Unit, Wellcome Trust-MRC Institute of Metabolic Science, University of Cambridge, Cambridge Biomedical Campus, Cambridge, CB2 0QQ UK

**Keywords:** Lipidomics, Prognostic markers, Gestational diabetes

## Abstract

We tested the hypothesis that both postnatal feeding and conditions *in utero* affect lipid metabolism in infants. Infants who experienced restrictive growth conditions *in utero* and others exposed to maternal hyperglycaemia were compared to a control group with respect to feeding mode. Dried blood spots were collected from a pilot subset of infant participants of the Cambridge Baby Growth Study at 3mo. Groups: (a) a normal gestation (control, *n* = 40), (b) small for gestational age (SGA, *n* = 34) and (c) whose mothers developed hyperglycaemia (*n* = 59). These groups were further stratified by feeding mode; breastfed, formula-fed or received a mixed intake. Their phospholipid, glyceride and sterol fractions were profiled using direct infusion mass spectrometry. Statistical tests were used to identify molecular species that indicated differences in lipid metabolism. The abundance of several phospholipids identified by multivariate analysis, PC(34:1), PC(34:2) and PC-O(34:1), was 30–100% higher across all experimental groups. SM(39:1) was around half as abundant in *in utero* groups among breastfed infants only. The evidence from this pilot study shows that phospholipid metabolism is modulated by both conditions *in utero* and postnatal feeding in a cohort of 133 Caucasian infants, three months *post partum*.

## Introduction

There is mounting evidence that conditions *in utero* can have profound and long-lasting effects on infant growth and metabolism. Recent studies in animal models have indicated that maternal diet and obesity are associated with cardio-vascular dysfunction^1,2^, inflammation of adipose tissue^3^, insulin resistance^4–6^, adiposity^5^, non-alcoholic fatty liver disease^7^ and even modulated nutrient acquisition^8^ in their offspring. Exercise has also been shown to rescue obese mothers’ insulin sensitivity and that of her male offspring^9^.

Similarly, a variety of exposures during pregnancy have been associated with birth outcomes in humans. It has been known for some time that factors such as smoking and a high caffeine intake during gestation lead to a lower birth weight in humans^10–12^. Both maternal obesity^13–15^ and gestational diabetes^16–19^ in humans typically lead to a higher birth weight, and higher rate of stillbirth^20,21^. Furthermore, babies small and large for gestational age are at increased risk of metabolic complications in later life^22,23^, which is consistent with the evidence from animal models of diet-induced obesity and gestational diabetes mellitus (GDM)^1–3,5^.

However, it is also becoming evident that dietary exposure in early life can have a profound effect on infant development in humans. Studies of the lipid profile in infant circulation show that this reflects feeding and correlates with development in the first years^24–26^, and hints that lipid metabolism is linked to infant development. This raises questions about how dysregulation of metabolism later in life is associated with conditions *in utero*, and how it might be detected early in life in order to facilitate the timing of appropriate intervention.

The importance of these questions is underscored by epidemiological evidence that the maternal hyperglycaemia (MHG) associated with GDM affects 8–24% of pregnancies in the UK, and is rising^27^. The concentration of glucose in the circulation of women who develop GDM is typically abnormally high due to decreased sensitivity to insulin and impaired insulin secretion. This results in higher placental transfer of glucose can contribute to more rapid growth of the foetus *in utero*. By contrast, there continue to be large numbers of babies who are born at a low birth weight. ‘Small for gestational age’ (SGA), based on sex and gestation-corrected size at birth, is often used to identify babies who had poor growth *in utero*. Several pregnancy-related maternal and placental factors may be involved in an infant falling into this group^28,29^, and such infants usually show rapid growth after birth^30^. Although much phenotypical data about both SGA and MHG infants has been collected, the metabolism driving these phenotypes is not clear.

This is particularly striking as several phospholipids in infant circulation correlate with their growth^24,25^. This raises questions about how infants of normal and high birth weight from GDM pregnancies differ, and whether the metabolism that arises from all normal-weight births are similar. The combination of the risks to health of both low- and high-weight births, the evidence that the phospholipid and triglyceride profiles in the circulation of infants is heavily dependent upon how they are fed^24,25^ and correlate with their growth, and evidence that conditions *in utero* shape infant growth, led us to the hypothesis that the metabolic response to diet in early life is modified by exposures *in utero*.

To address this question, we assessed the circulating phospholipid and triglyceride profile of infants from three groups stratified by conditions *in utero*, and within each group we compared the mode of feeding (breastfed, formula-fed, or mixed-fed). The three *in utero* groups of infants included were (a) born at low birth weight for gestational age (SGA), (b) exposed to maternal hyperglycaemia (MHG) but with a normal birth weight, or (c) Controls (normal birth weight and no MHG). The groups were otherwise homogeneous, none of the mothers of SGA babies had hyperglycaemia. Each group comprised individuals who were breastfed, formula-fed or received a mixed intake. This allowed us to compare the *in utero* exposure across different *post natal* (first 3mo of life) feeding types, and also feeding types across infants exposed to different conditions *in utero*. This was designed to give an insight into the way both *post natal* (first 3mo of life) feeding and conditions *in utero* affected the observed lipid metabolism at three months of age. The method for profiling the phospholipids and triglycerides present (mass spectrometry) was chosen to identify individual metabolites in order to characterise differences in lipid metabolism.

## Results

### Augmentation of reported data

In order to identify all possible lipid/triglyceride variables that may be associated with the phenotypical or diagnostic difference between groups, we judged that molecular species identified in both positive and negative mode should be used. We therefore augmented data from a previous study, that was based only on positive ionisation mode^24,25^, with the appropriate lipid profiles from signals collected in negative ionisation mode. Statistical tests (Principal component analysis followed by T-tests) performed on this augmented dataset showed that it exhibits the same trends as the positive mode data alone; there is a clear separation between the phospholipid and glyceride profiles of the three feeding groups tested (breastfed infants, formula-fed infants and infants receiving a mixed intake), with the mixed feeding group lying between the other two (Supplementary Fig. 1A). We then examined the abundance of biomarkers of feeding identified previously in additional infants, who form the normal birth weight Control group of the current study, Supplementary Fig. 1B. This approach is used to validate the control group of this study with that used in previous work. Both the magnitude and pattern of abundance of these biomarkers of feeding type appears to be similar in this independent Control group (Supplementary Fig. 2).

### Influence of feeding mode across in utero groups

The hypothesis of this study is that both postnatal feeding and conditions *in utero* influence the lipid metabolism observed in infants at 3mo. In order to test this, we first stratified the participants only by the *in utero* groups, *i*.*e*. those who were small for gestational age (SGA), those who were exposed to maternal hyperglycaemia *in utero* and those of healthy pregnancies, and sought to identify the variables that distinguish the groups without reference to how the participants were fed. A Random Forest classification found no molecular species that distinguished the *utero* groups (SGA, MHG, Control). This suggests that feeding mode has a more pronounced influence on the lipids *in circulo* and thus we stratified the participants by the feeding mode as well as by conditions *in utero*. This approach allows us to distinguish any effects of conditions *in utero* from those of feeding mode.

### Stratification of the participants by both feeding type and conditions *in utero*

The evidence that the *in utero* groups alone did not dominate the lipid metabolism at 3mo led us to stratify the infants according to feeding type (breastfed, mixed, formula-fed) as well as the conditions they were experienced to *in utero* (giving rise to the SGA, MHG and control groups). This gave nine groups. In order to test whether lipid metabolism was different in the exposed groups (SGA, MHG), we compared exposed and control groups of the same feeding type. A supervised statistical test (sPLS-DA) was used to identify (a) the individual variables (molecular species) that distinguished, for example, the breastfed MHG group from the breastfed Control group and (b) the feeding groups within the exposed groups (*e*.*g*. breast and formula fed individuals from the MHG group). This structure was adopted in order to compare individuals who differed in either conditions *in utero* or by feeding mode.

### Variables indicating shifts in lipid metabolism in SGA and MHG infants

None of the species identified in previous work^25^ was identified as a variable that drove the distinctions between different feeding types in SGA infants (Supplementary Fig. 3A, loading values in table). The lipid species that were identified as distinguishing the breastfed from formula fed SGA infants (SM(36:2) and SM(34:2)) were structurally similar to those found previously. Several lipid species distinguish MHG infants from controls (Table 1). However, three of these variables are also identified as the three most important variables for distinguishing the three feeding groups of MHG infants from one another: PC(34:2), SM(32:1) and SM(39:1) (Supplementary Fig. 3B).

**Table 1:** Loading values for candidate biomarkers.

Lipid	SGA						MHG					
	Breast-fed		Mixed-fed		Formula-fed		Breast-fed		Mixed-fed		Formula-fed	
	PC1	PC2	PC1	PC2	PC1	PC2	PC1	PC2	PC1	PC2	PC1	PC2
PC(34:1) & PE(37:1)	0.532	−0.176	0.599	−0.254	0.549	−0.133	0.566	−0.254	0.597	−0.269	0.573	−0.268
PC(34:2)	0.489	0.230	0.356	−0.095	0.403	−0.312	0.389	−0.028	0.450	−0.094	0.385	−0.290
PC(38:4) & PE(41:4)	0.041	−0.030	0.039	−0.032	0.034	−0.031	0.049	−0.006	0.053	0.104	0.003	−0.041
PC-O(34:1) & PC-P(34:0)	0.020	−0.015	0.023	−0.013	0.021	0.002	0.022	−0.017	0.025	−0.012	0.022	−0.006
SM(36:2)	0.015	−0.010	0.006	−0.002	0.008	−0.003	0.014	0.001	0.008	0.012	0.007	0.009
SM(34:2)	0.014	0.000	0.001	0.009	0.002	0.010	0.009	0.010	0.006	0.015	0.008	0.019
SM(32:1)	0.008	−0.036	0.014	0.039	0.001	0.061	0.009	0.005	0.020	0.020	0.012	0.018
PC-O(36:4) & PC-P(36:3)	0.003	−0.002	0.001	−0.001	0.004	−0.004	0.005	−0.004	0.007	0.009	0.002	0.006
SM(39:1)	−0.009	−0.008	−0.011	0.011	−0.013	0.016	−0.007	0.001	−0.006	0.005	−0.009	0.017

Loading values generated by PCAs for the candidate biomarkers identified by sPLS-DA. Each exposed group (*e.g.* SGA-breastfed) tested against the appropriate control group (*e.g.* Control-breastfed). Loading values reflect the ranking of the variables by the (unsupervised) test. Note that the quantified rankings are only valid within principal components and thus cannot be compared between the groups. This test indicated that similar variables to those identified in Fig. 2 were important.

### Identification of candidate biomarkers (CBMs)

**Figure 1:**
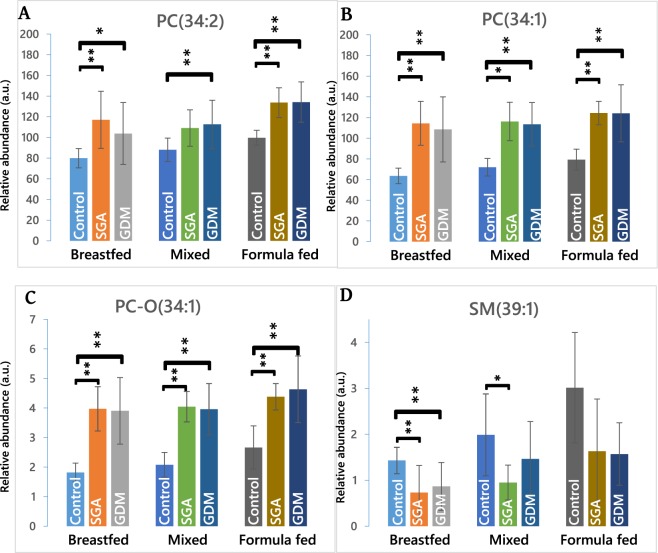
The abundance of candidate biomarkers in infants with normal conditions in utero as well as in SGA and MHG. Only these four lipids passed FDR based on a Bonferroni adjusted significance threshold. Panel (**A**) PC(34:2), isobaric with PE(37:2); (**B**) PC(34:1), isobaric with PE(37:1); (**C**) PC-O(34:1), isobaric with PC-P(34:0); (**D**) SM(39:1). No. variables = 340. *p*-Values for Bonferroni corrected T-test: for dependent variables = 0·00271 (marked*) and independent variables = 0·000147 (marked**) respectively.

In order to identify CBMs for differences in lipid metabolism from the variables identified by the sPLS-DA, the abundance of the molecular species must also be considered. The abundances of the variables across the various groups were calculated (mean of all values in each group) and T-tests applied in order to quantify the significance of the difference in the mean of abundance in exposed and control groups. The *p*-value cut offs used were 0·00271 for dependent variables and 0·000147 for independent variables. These tests identified PC(34:1), PC(34:2) and PC-O(34:1) particularly clearly (Fig. 1). The abundance of these phospholipids was higher in MHG and SGA groups compared to Controls, howsoever fed (Fig. 1 and Supplementary Table 1). SM(39:1) showed differences between *in utero* groups among breastfed infants, but these differences were not significant among mixed-fed or formula-fed infants. Among breast-fed infants only, differences between the three *in utero* groups were also seen in the abundance of PC(38:4) and SM(36:2), but these were not significant according to the Bonferroni adjusted threshold.

**Figure 2:**
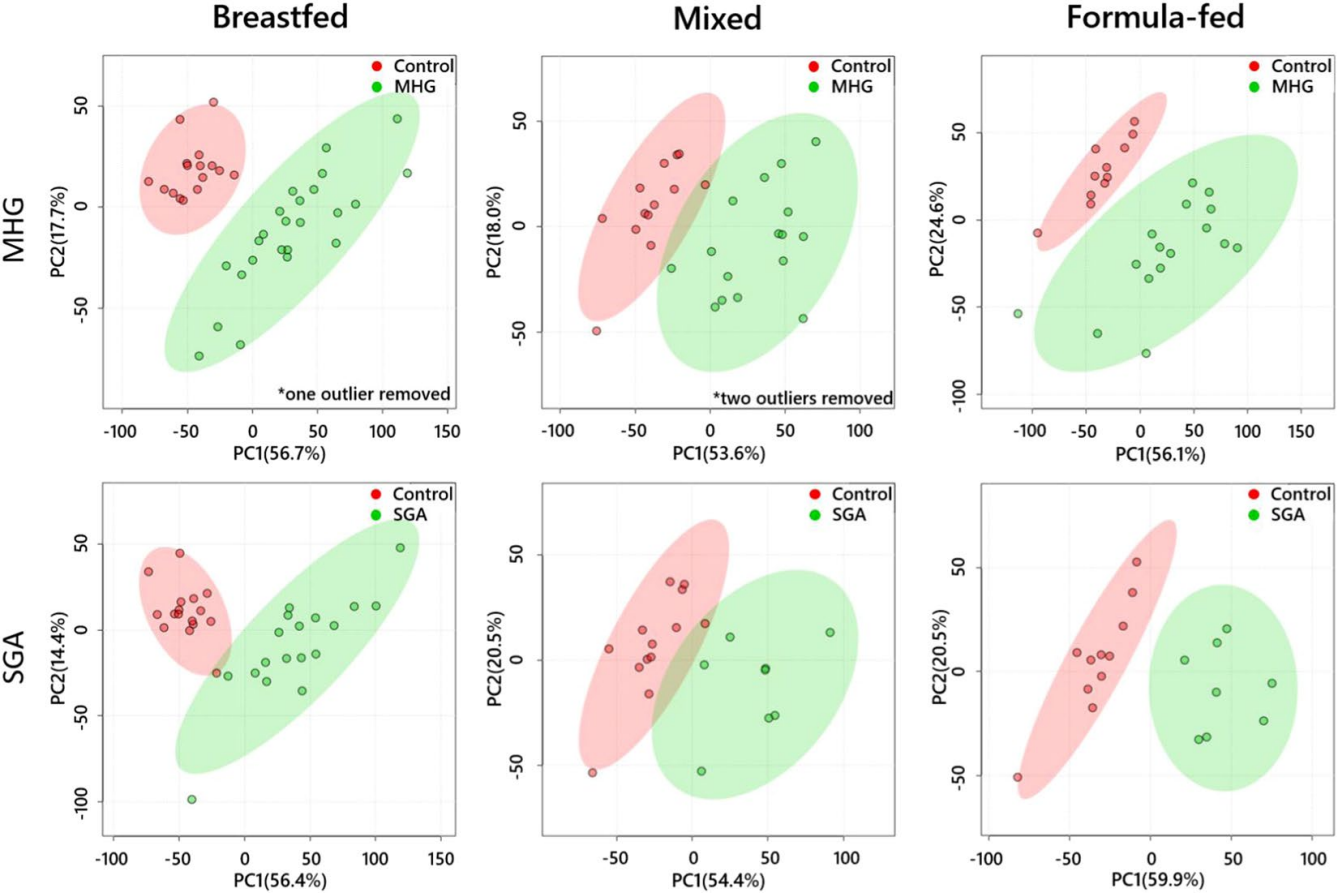
Principal Component Analyses of lipid profiles of infants born as SGA or from mothers with gestational diabetes (green) against controls (red).

Notably, the abundances of PC(34:1), PC(34:2) and PC-O(34:1) were higher in both SGA and MHG compared to controls (Fig. 1). By contrast, the abundance of SM(39:1) is lower in SGA and MHG infants than in controls. In order to verify the results of the sPLS-DA and univariate tests, we performed an unsupervised analytical approach (principal component analysis, PCA) on the whole dataset. The PCAs (Fig. 2) showed that all subgroups of SGA and MHG infants were distinct from Control infants, across all feeding types. Furthermore, PC(34:1) and PC(34:2) are the most important of these CBMs in distinguishing the groups, as they were more abundant than any other species measured (Fig. 1, full loadings shown in Supplementary Table 2).

## Discussion

In this study, mass spectrometry was used to profile the phospholipids and triglycerides in the circulation of infants; statistical approaches were used to identify firstly variables and after more thorough testing candidate biomarkers (CBMs), that distinguish breastfed infants from those formula fed and conditions *in utero* simultaneously. These approaches identified PC(34:1) and PC(34:2) as the CBMs that represent the clearest difference in phospholipid metabolism, with PC-O(34:1) and SM(39:1) performing more moderately, but also being significant. However, all four of these lipids have the same head group (choline) and three of the four are very similar isoforms (34:1 or 34:2). This uniformity hints that these species may originate from a common process or under control of the same pathway. These species undoubtedly have structural roles *in vivo*, however, PCs are increasingly being recognised as having signalling functions as well, including these isoforms of PC^31^. In addition, SM(39:1) has recently been identified as a marker for dietary intake of fresh milk^24^. The PCs were more abundant in the circulation of infants from abnormal uterine conditions (SGA and MHG) than in Controls, whereas SM(39:1) was less abundant than controls across all feeding types.

Evidence collected over the last decade has shown that an isoform of PC(34:1), PC(16:0/18:1), has been identified as an endogenous ligand (agonist) for the nuclear receptor PPARα (proxisome proliferator-activated receptor alpha), found in hepatocytes. PPARα is a transcription factor regulating the expression of a number of genes involved in governing metabolism of triglycerides^32^ whose expression increases in foetal liver in pregnancies that progress normally^33^. Activation of PPARα is observed under fasting conditions^34^ and is characterised by increases in the catabolism of fatty acids and the abundance of peroxisomes^32^. In adults, it also decreases hepatic steatosis^35^ but accelerates the development of the epidermal permeability barrier in the foetus^36^. PC(34:1) increases in abundance by up to 100% between the control and both MHG and SGA groups in this study (Fig. 1). This suggests that activation of PPARα may be more common in the exposed groups. If true, one would therefore expect a faster rate of catabolism of fatty acids, and a weaker tendency of them being stored as triglycerides in SGA and MHG babies. An interaction such as this may form the basis for the metabolic programming effects observed by the conditions *in utero* and the feeding type afterwards.

We note that PC(34:1) appears as both a *post natal* nutritional CBM (*i*.*e*. for feeding type) and one that distinguishes conditions *in utero*. It is not clear from the present evidence whether this lipid is ubiquitous or has several functions. There is a plethora of evidence for a variety of phospholipids possessing even three or more functions simultaneously^31,37,38^. One possibility is that the structural differences between the control and exposed groups in this study have driven changes in the abundance of commonplace structural molecules such as PC, that also have other roles^31^.

The abundance of PC(18:0/18:1), an isoform of PC(36:1), is also different in breastfed infants in this study. The abundance of PC(36:1) is around 60% higher in both SGA and MHG groups than normal birth weight Controls, among infants receiving breastfeeding (Table 3). However, the abundance is similar in across *in utero* groups fed formula (Table 3). PC(18:0/18:1) is an endogenous agonist for another PPAR, PPARδ, with activation reducing the concentration of triglycerides in plasma *post prandium* and increasing fatty acid use in the periphery^39^. However, there is also evidence that the two receptors are sufficiently specific such that PC(18:0/18:1) is not cross-reactivate with the receptor specific for PC(16:0/18:1)^31^.

These data therefore suggest that activation of PPAR_δ_ may be different in babies receiving formula from those receiving breastmilk. If true, the triglycerides consumed by babies ingesting breastmilk will have a shorter half-life (be in the circulation for a shorter time) than those only fed formulae.

Furthermore, a naturally-occurring, endogenously-produced oxidised form of PC-O(34:1) is a potent agonist of PPARγ^40^. PPARγ is widely regarded as a key transcription factor in adipocyte differentiation^41,42^ and as PC-O(34:1) is much more abundant in infants from GDM pregnancies than controls, we might predict that more of the oxidised form (oxPC-O(34:1), which is commercially available) *in circulo* and that this leads to higher activation of PPARγ in SGA and MHG infants. This is significant because activation of PPARγ through an increase in the abundance of PC-O(34:1) provides a mechanism by which this species promotes adiposity. This may be important in long term metabolic health as there is now evidence that adiposity in adolescents is modulated by breastfeeding countering the deleterious effects of a PPARγ^43^.

More generally, how PPARs are expressed in the foetus can be influenced by maternal diet and obesity^44,45^. Specifically, an animal model has been shown that intra-uterine growth restriction decreases the expression of PPARγ in the lung^46^ but increases it in adipocytes^47^.

The summative effect of both the putative increased abundance of agonists for PPARα, PPARδ and PPARγ and changes to their expression is unclear at present, however we expect a mixture of effects with respect to healthy systems as the catabolism of fatty acids and storage of TGs is favoured. This has implications for the relationship between conditions *in utero* and shortly thereafter, and later development. Further work is required to establish whether the CBMs identified in this study have a functional or metabolic programming influence on metabolism in infants who experienced different conditions *in utero*. This could be achieved by another study with increased statistical power but also follow-up samples and *meta*-data. Further work is also required to determine the origin and an understanding of how the abundance of such species is controlled, either by the infant’s system or the maternal one.

The sPLS-DAs used to identify CBMs can also be used to assess how similar the individual members of the groups are to one another. For example, the formula-fed infants in both MHG and SGA groups appear to be far more homogeneous than either the mixed or breastfed groups (Supplementary Fig. 3). This raises questions about the variety of phospholipid and triglyceride profiles in breastmilk, and their relationship with the mothers’ diet. A larger sample size in a different group of individuals is required to investigate this. However, these results suggest that the metabolic activity in formula-fed SGA and MHG infants is more homogeneous than other groups, hinting that a narrower range of factors shapes the lipid profile found in their circulation.

Lastly, recent work on the lipid and triglyceride composition of infant formula suggests that this type of dietary intake varies considerably by manufacturer, manufacturing site and target demographic^48^. This may explain some of the heterogeneity in the groups fed formula. Several of the most abundant phospholipid species were similar across formulae, though the triglyceride profiles of formula and fresh milk were distinct. This suggests that further study of the relationship between lipid metabolism following the feeding of infant formula, and in a high powered manner, is required.

The present study represents a pilot, mechanistic-type investigation of the relationship between conditions *in utero* and later feeding. To our knowledge it is the first of its kind. It is limited in the manner that is typical of pilot studies (*e*.*g*. modest statistical power). Recent work on infant formula has shown that it is a varied and complicated mixture, suggesting that the traditional classification of ‘formula-fed’ used in the present study and many others is a general one. However, the present study is capable of suggesting which of the comparisons are more important and thus which are of principal interest for further studies, both clinical and mechanistic.

## Conclusion

In this study, the hypothesis that the lipid and triglyceride metabolism in infants is shaped by antenatal conditions (including glucose supply) and postnatal nutrition was tested in a pilot cohort by profiling the phospholipid, triglyceride and sterol fractions in the circulation of infants three months *post partum*. Three separate statistical methods indicate that the abundance of a small number of phospholipids with a narrow range of structures (PC34:1), PC(34:2) and PC-O(34:1)) best distinguishes the groups of individuals. We note that phospholipids with this molecular structure have an important role in triglyceride metabolism in humans, through interaction with PPARs. Furthermore, the evidence from the separate analyses in this study indicates that the feeding type applied to infants is as important for the phospholipid profile observed in infants at three months *post partum* as the effect of the in uterine conditions associated with SGA and MHG. This study therefore provides evidence that both feeding and conditions *in utero* shape short-term phospholipid metabolism in early life. Furthermore, the similar nature of the pattern of abundance of CBMs between exposed groups (SGA and MHG) indicate that the shifts in lipid metabolism may be similar between these groups.

The precise role or effects in infants of the candidate biomarkers identified in this study is not yet fully understood. Further work may explore how changes in phospholipid metabolism can be used to correct the metabolic consequences of unfavourable conditions *in utero*.

## Experimental Methods

### Study design

This pilot study was designed to determine what, if any, differences in phospholipid, sterol and/or triglyceride metabolism there are between infants as a factor of both conditions in utero and feeding in the first three months of life. This necessitated a suitable array of participants, consistent sample collection, detailed measurement of molecular abundance and statistical analyses.

### Cohort

We used a subset of infant participants from the Cambridge Baby Growth Study II, characterised in Table 2. Only samples from singleton births were used. This is a prospective observational birth cohort, focussing on the antenatal and early postnatal determinants of infancy growth (22) Mothers were recruited during early pregnancy from a single antenatal centre in Cambridge.

**Table 2:** Participant characteristics.

		Control	SGA	MHG
*n*		40	34	59
	Breastfed	16	18	25
	Mixed feeding	13	8	18
	Formula fed	11	8	16
Birth weight (Kg)		39	34	59
	Mean	3.37	2.41	3.3
	s.d.	0.38	0.24	0.37
	Range (Lowest)	2.65	1.86	2.72
	Range (Highest)	4.02	2.85	4.88
	Significance			
	against control group	—	5.10E-09	0.63
	against SGA group	5.10E-09	—	5.10E-09
	against MHG group	0.63	5.10E-09	—
Gestational age (w)		40	33	59
	Mean	39.84	39.64	39.05
	s.d.	1.33	1.61	0.94
	Range (Lowest)	36.00	36.14	36.57
	Range (Highest)	41.86	43.00	41.43
	Significance			
	against control group	—	0.76	7.04E-03
	against SGA group	0.76	—	0.08
	against MHG group	7.04E-03	0.08	—
No. male (%)	Breastfed	31	50	32
	Mixed feeding	62	38	78
	Formula-fed	27	38	88
Maternal age (years)		40	33	59
	Mean	32.89	33.19	34.53
	s.d.	5.42	4.68	4.34
	Range (Lowest)	19.53	23.15	22.69
	Range (Highest)	41.33	43.56	44.14
	Significance			
	against control group	—	0.96	0.22
	against SGA group	0.96	—	0.40
	against MHG group	0.22	0.40	—
Maternal BMI		34	30	46
	Mean	22.77	24.1	25.92
	s.d.	4.29	4.88	6.05
	Range (Lowest)	17.68	19.22	17.33
	Range (Highest)	35.23	35.48	44.39
	Significance			
	against control group	—	0.57	0.02
	against SGA group	0.57	—	0.30
	against MHG group	0.02	0.30	—

The characteristics of the participants of the subset if the Cambridge Baby Growth Study II cohort used in this study. *n* = 133. Significance was calculated using a Tukey HSD post-hoc analysis. Maternal BMI was taken at or before conception. Maternal age is as at delivery.

**Table 3:** Abundance of PC(36:1).

	Mean	StDev	T-test	Loadings values	
				PC1	PC2
Control (BF)	14·072	2·250			
SGA (BF)	25·106	4·530	1·573 × 10^−9^	0·1026	−0·0862
MHG (BF)	25·070	7·010	6·370 × 10^−8^	0·1270	−0·0833
Control (M)	15·099	2·054			
SGA (M)	26·355	3·995	3·440 × 10^−5^	0·1356	−0·0806
MHG (M)	24·438	4·271	1·545 × 10^−8^	0·1189	−0·0754
Control (FF)	15·415	2·706			
SGA (FF)	14·230	2·172	3·409 × 10^−7^	0·1284	0·0072
MHG (FF)	14·072	2·250	1·701 × 10^−8^	0·0947	−0·0588

Mean and standard deviation of abundance of PC(36:1), p-value associated with the difference between exposed and control samples and the loadings in the PCAs (Fig. 2). PC(36:1) is not a CBM due to the similar abundance across formula fed groups. BF, breastfed; M, mixed feeding; FF, formula-fed.

There were three groups of infant participants according to conditions *in utero*: (a) infants being born small for gestational age (SGA group), (b) a normal birth weight (Control group) and (c) a normal birth weight group who had exposed to maternal hyperglycaemia (MHG group; mothers of infants in this group were diagnosed according to IADPSG thresholds for GDM)^49^. There were three groups within each of these *in utero* groups, based on feeding mode. Feeding groups were of breastfed, formula-fed or infants receiving a mixed intake (Table 2). We were not able to determine the relative proportions of the two due to well-known experimental difficulties in establishing the volume of milk consumed during breast feeding.

### Ethics

All procedures were performed in accordance with the ethical standards of the institutional and/or national research committee and with the 1964 Helsinki declaration and its later amendments or comparable ethical standards. All mothers gave informed written consent and the study was approved by the Cambridge local research ethics committee (LREC 11/EE/0068). Class membership of samples were blinded and so technicians and analysts were not aware of which participants fell into which groups until after the lipid profiles were available, and then only by participant number.

### Reagents and standards

Solvents of at least HPLC grade were purchased from *Sigma-Aldrich Ltd*. (Gillingham, Dorset, UK) and were not purified further. Lipid standards were purchased from Avanti Polar lipids (Alabaster, AL; through Instruchemie, Delfzijl, NL) and used without purification. Consumables were purchased from Sarstedt AG & Co (Leicester, UK).

### Sample collection

Samples from the three *in utero* groups were drawn from infants (*n* = 133) who participated in the *Cambridge Baby Growth Study II* and collected between 2011 and 2013. Their infants were seen at birth by trained research nurses and followed-up at 3 months. Blood was collected by a heel prick, spotted immediately on untreated filter paper cards (Ahlstrom 226; ID Biological Systems, Greenville, South Carolina) and dried at room temperature overnight. Dried blood spots were stored under a small, sealed air atmosphere at −80 °C. Samples were collected during morning appointments (0900–1130). Infancy feeding (exclusive breast-, mixed or exclusive formula-feeding) was assessed by questionnaire at age 3 months.

### Lipid extraction

The phospholipid, triglyceride and sterol fractions were isolated together using a method reported recently^50^. Briefly, dried blood spot samples, as 3·2 mm diameter discs, were placed along with blank and Quality Control samples (QCs) in the wells of a glass coated 2.4 mL/well ninety-six-well plate (96w plate; Plate+™, Esslab, Hadleigh, UK). Water (100 μL, MilliQ) was added to each of the wells, followed by methanol (250 μL, HPLC grade, spiked with 0·6 μM 1,2-di-*O*-octadecyl-*sn*-glycero-3-phosphocholine, 1·2 μM 1,2-di-*O*-phytanyl-*sn*-glycero-3-phosphoethanolamine, 0·6 μM C_8_-ceramide, 0·6 μM *N*-heptadecanoyl-d-erythro-sphingosylphosporylcholine, 6·2 μM undecanoic acid, 0·6 μM trilaurin), followed by *tert*-butyl methyl ether (TMBE, 500 μL). The plates were then sealed (aluminium microplate sealing tape), agitated (10 min, 600 rpm) and centrifuged for 10 min at 3·2 k × *g*. A 96-head micro-dispenser was used to transfer 200 μL of the organic solution to a glass-coated 240 μL 96w plate (Plate+™, Esslab, Hadleigh, UK). The plate was transferred to a Genevac EZ-2 evaporator (Genevac Ltd., Ipswich, UK) and dried. The samples were reconstituted (TBME, 25 μL and MS-mix [7.5 mM ammonium acetate in IPA:CH_3_OH (2:1)], 90 μL) of using a Hydra Matrix, after which the plate was sealed and stored at −20 °C.

### Mass spectrometry

All samples were infused into an Exactive Orbitrap (Thermo, Hemel Hampstead, UK), using a Triversa Nanomate (Advion, Ithaca US) and a mass resolution of 100,000. Samples were ionised at 1·2 kV. The Exactive started acquiring data 20 s after sample aspiration began. After 72 s of acquisition in positive mode the Nanomate and the Exactive switched to negative mode, decreasing the voltage to −1·5 kV. The spray was maintained for another 66 s, after which the analysis was stopped and the tip discarded, before the analysis of the next sample began. Throughout the analysis the sample plate was kept at 15 °C. Samples were run in row order.

The phospholipid and triglyceride signals obtained were relative abundance (‘semi-quantitative’), with the signal intensity of each lipid expressed relative to the total lipid signal intensity, for each individual, per cent (%). Raw high-resolution mass-spectrometry data were processed using XCMS (www.bioconductor.org) and Peakpicker v 2.0 (an in-house R script).

### Statistical methods

In order to identify which individual molecular species best represented the differences in the lipid metabolism between the various groups, we employed several statistical methods. All multivariate analyses (MVA) except Random Forest were carried out using Metaboanalyst 4.0^51^. Random Forest classifications were carried out using R. Univariate tests were carried out using Microsoft Excel 2013. Significance thresholds were corrected for multiple testing and *p*-value cut offs used were 0·00271 for dependent variables and 0·000147 for independent variables.

The first stage of our analysis was to augment a previous analysis with the lipid profile collected in negative ionisation mode. The previous analysis used species detected in positive mode only^25^. This employed the same unsupervised test used in previous work, *viz*. a Principal Component Analysis (PCA). This indicated a fuller range of detectable molecular species that were associated with feeding type. The second stage was designed to test whether conditions *in utero* (SGA, MHG, Control) had an effect on lipid metabolism that was generally independent of that of feeding. A Random Forest classification was used for this test, using three groups and 10k trees. The third stage was candidate biomarker discovery. This comprised two parts. First, a sparse Partial Least Squares Discriminant Analysis was used to identify individual variables that distinguished the groups from one another, followed by (univariate) T-tests to identify which candidate biomarkers (CBMs) were significantly different in abundance. Second, an unsupervised MVA statistical test was used to verify the ranked importance of the CBMs identified in the first stage (supervised MVA and univariate tests). The type of MVA was unsupervised (PCA) because this offers an orthogonal approach to identifying variables but also to minimise bias.

## Supplementary information

Supplementary Information

## Data Availability

The datasets generated during and analysed during the current study are available from the corresponding author on reasonable request.
