# Supplementary material for: Evidence from 3-month-old infants shows that a combination of postnatal feeding and exposures in utero shape lipid metabolism
Source: Sci Rep. 2019 Oct 4;9:14321. doi: 10.1038/s41598-019-50693-0 (PMC6778076; doi:10.1038/s41598-019-50693-0)
Supplement: Supplementary file 1 — Supplementary Information [file 41598_2019_50693_MOESM1_ESM.docx]

Supplementary information for:

Evidence from 3-month-old infants shows that a combination of postnatal feeding and exposures *in utero* shape lipid metabolism

*Samuel Furse­^1^, Stuart G. Snowden­^1^, Laurentya Olga^2^, Philippa Prentice^2^, Ken K. Ong^2,3^, Ieuan A. Hughes^2^, Carlo L. Acerini^2^, David B. Dunger^2^, Albert Koulman^1,3, *^*

*^1^Core Metabolomics and Lipidomics Laboratory, Metabolic Research Laboratories, Institute of Metabolic Science, University of Cambridge, Level 4 Pathology, Cambridge Biomedical Campus, Cambridge CB2 0QQ, UK.*

*^2^Department of Paediatrics, University of Cambridge, Box 116, Cambridge Biomedical Campus, Cambridge CB2 0QQ, U.K.*

*^3^MRC Epidemiology Unit, Wellcome Trust-MRC Institute of Metabolic Science, University of Cambridge, Cambridge Biomedical Campus, Cambridge, CB2 0QQ, UK.*

Supplementary data – Figure S1


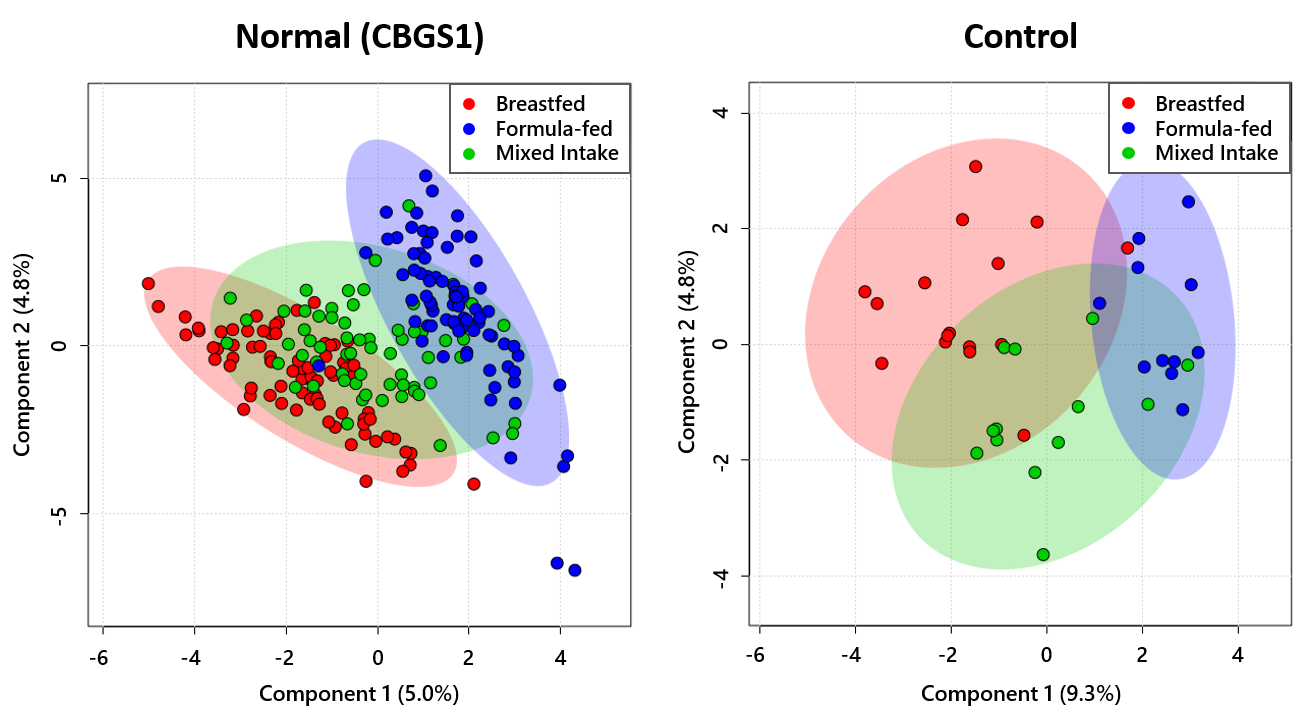


**B.**

**A.**

| Lipid | Component 1 | Component 2 |  | Lipid | Component 1 | Component 2 |
| --- | --- | --- | --- | --- | --- | --- |
| **SM(32:1)** | **0·0293** | **0·0275** |  | **PC(34:2)** | **0·5214** | **0** |
| **SM(39:1)** | **0·2593** | **0** |  | **SM(39:1)** | **0·2661** | **0** |
| PC-P(34:1) & PC-O(34:2) | 0 | 0·5659 |  | **PC(34:1) & PE(37:1)** | **0·1742** | **0** |
| PC-O(34:1) & PC-P(34:0)* | 0 | 0·5048 |  | SM(38:1) | 0·1709 | 0 |
| **PC(34:2)** | **0** | **0·3761** |  | **SM(32:1)** | **0·1120** | **0** |
| PC-O(36:3) & PC-P(36:2) | 0 | 0·3533 |  | PS(31:1) | 0 | 0·5177 |
| **PC(34:1) & PE(37:1)** | **0** | **0·3120** |  | DG(34:3) | 0 | 0·0744 |
| PC(34:3) & PE(37:3) | 0 | 0·2088 |  | DG(36:4) | 0 | 0·0552 |
| PC(40:4) & PE(43:4) | 0 | 0·1222 |  | TG(36:3) | 0 | 0·0092 |

*Figure S1. sPLS-DA of the lipid profiles of infants 3 months* post partum*, stratified by feeding type (1, Breastfed; 2, mixed intake; 3, formula-fed). A, participants from CBGS I (data collected form positive ion mode already reported^2^); B, Control group used for this study (n = 40). Lipids in bold type appear in both analyses. *also appears in previous work^2^.*

Supplementary data – Figure S2

**A.**

**B.**

**B.**

*Figure S2. The relative abundance of candidate biomarkers for infant development identified in previous work (Prentice* et al.*, 2015) in both the original samples (augmented with data collected in negative ion mode) and independent normal birth weight Controls (positive and negative ion modes).*

Supplementary data – Figure S3


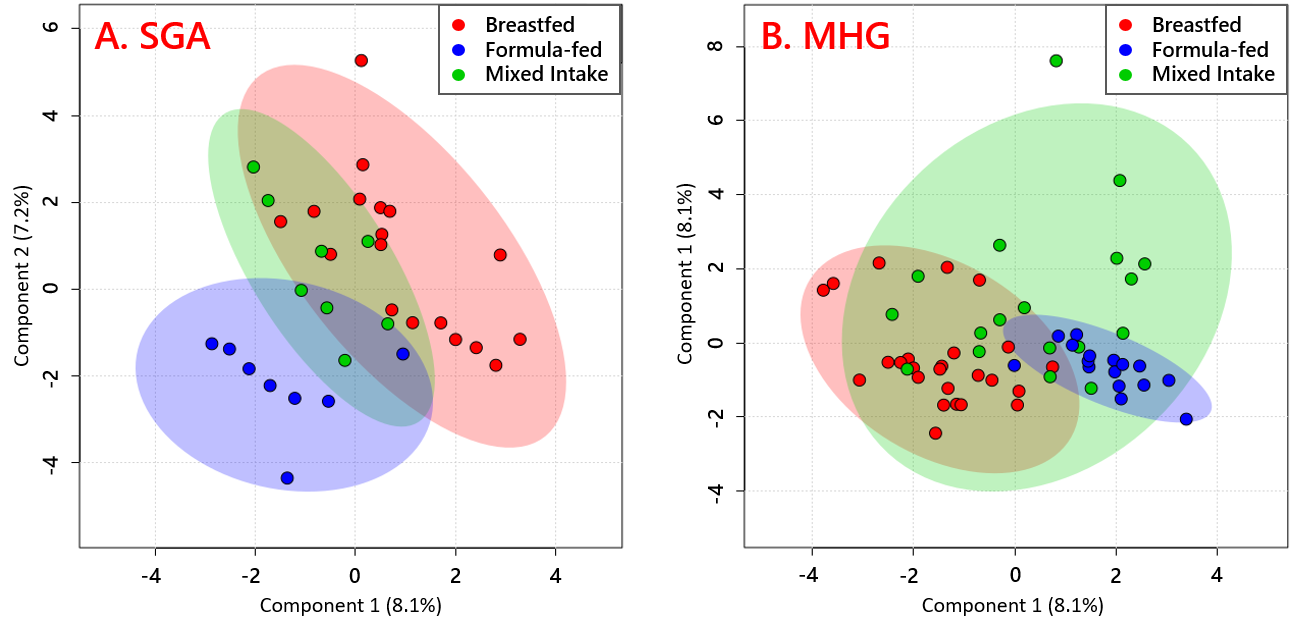


| Lipid | Component 1 | Component 2 |  | Lipid | Component 1 | Component 2 |
| --- | --- | --- | --- | --- | --- | --- |
| SM(36:2) ^1^ | 0·8908 | 0 |  | **SM(32:1)** | 0·3536 | 0 |
| SM(34:2) ^1^ | 0·3121 | 0 |  | **SM(39:1)** | 0·1495 | 0 |
| SM(42:3) | 0·1436 | 0 |  | **PC(34:2)** | 0·1037 | 0 |
| PC(35:2) | 0·0504 | 0 |  | SM(40:3) | 0 | 0·4448 |
| PC(38:5) & PE(41:5) | 0·0400 | 0 |  | PIP(52:2) | 0 | 0·4335 |
| PE(40:3) & PC(37:3) | 0·0375 | 0 |  | PI-5-*P*(17:0/20:4) | 0 | 0·3556 |
| PC(38:3) & PE(41:3) | 0·0155 | 0 |  | TG(56:2) | 0 | 0·2644 |
| Cer(42:1) | 0 | 0·1038 |  | TG(50:4) | 0 | 0·2552 |
| TG(50:1) | 0 | 0·0907 |  | PA(18:4/13:0) | 0 | 0·1853 |

*Figure S3. sPLS-DA of the lipid profiles of infants 3 months* post partum*, stratified by feeding type (1, Breastfed; 2, mixed intake; 3, formula-fed). A, participants who were small for gestational age (SGA) at delivery; B, Infants delivered from mothers who developed gestational diabetes (MHG). Lipids in bold type also appear in analyses of Control infants* (Fig. S2 and S3)*.* ^1^*species carried forward in the targeted analysis.*

Supplementary Data

Reliability through *p*-values of the abundance of CBMs across experimental and control groups

|  | Breast-fed | | Mixed-fed | | Formula-fed | |
| --- | --- | --- | --- | --- | --- | --- |
|  | SGA | MHG | SGA | SGA | MHG | SGA |
| PC-O(34:1) & PC-P(34:0) ^1^ | **<0·0001** | **<0·0001** | **<0·0001** | **<0·0001** | **<0·0001** | **<0·0001** |
| PC(34:1) & PE(37:1) ^1,2^ | **<0·0001** | **<0·0001** | **<0·0001** | **<0·0001** | **<0·0001** | **<0·0001** |
| PC(34:2) ^2^ | **<0·0001** | **0·0008** | 0·012 | **<0·0001** | **0·0008** | 0·012 |
| SM(39:1) ^2^ | **0·0001** | **0·0001** | **0·0017** | **0·0001** | **0·0001** | **0·0017** |
| PC(38:4) & PE(41:4) ^1^ | 0·0042 | 0·0076 | 0·018 | 0·0042 | 0·0076 | 0·018 |
| SM(36:2) ^2^ | 0·0082 | 0·013 | 0·80 | 0·0082 | 0·013 | 0·80 |
| PC-O(36:4) & PC-P(36:3) ^1^ | 0·10 | 0·029 | 0·31 | 0·10 | 0·029 | 0·31 |
| SM(32:1) ^2^ | 0·040 | 0·40 | 0·95 | 0·040 | 0·40 | 0·95 |
| SM(34:2) ^1^ | 0·085 | 0·95 | 0·16 | 0·085 | 0·95 | 0·16 |

*Table S1.* p*-Values from two-tailed t-tests for comparisons between small for gestational age (SGA) or maternal hyperglycaemia (MHG) infants versus Control infants (normal birth weight and non-MHG), stratified by feeding regime. n = 10-20 per group. ^1^CBM identified in previous work^2^; ^2^Candidate biomarkers of milk feeding regime (CBM) identified in the current study;. No. variables = 340. Bonferroni corrections (*p *= 0·05) for dependent variables = 0·00271 and independent variables = 0·000147.*

Loading values for PCAs shown in Figure S2.

|  | SGA (BF) | | SGA (M) | | SGA (FF) | | MHG (BF) | | MHG (M) | | MHG (FF) | |
| --- | --- | --- | --- | --- | --- | --- | --- | --- | --- | --- | --- | --- |
|  | PC1 | PC2 | PC1 | PC2 | PC1 | PC2 | PC1 | PC2 | PC1 | PC2 | PC1 | PC2 |
| PC(34:1) & PE(37:1) | 0.53 | -0.18 | 0.60 | -0.25 | 0.55 | -0.13 | 0.57 | -0.25 | 0.60 | -0.27 | 0.57 | -0.27 |
| PC(34:2) | 0.49 | 0.23 | 0.36 | -0.10 | 0.40 | -0.31 | 0.39 | -0.03 | 0.45 | -0.09 | 0.38 | -0.29 |
| PE(39:2) & PC(36:2) | 0.31 | 0.12 | 0.24 | -0.22 | 0.33 | -0.21 | 0.29 | -0.02 | 0.24 | -0.10 | 0.21 | -0.21 |
| PE(42:5) & PC(39:5) | 0.19 | 0.05 | 0.21 | -0.04 | 0.09 | -0.06 | 0.14 | 0.06 | 0.14 | 0.11 | 0.08 | -0.22 |
| PC(36:1) & PE(39:1) | 0.10 | -0.09 | 0.14 | -0.08 | 0.13 | 0.01 | 0.13 | -0.08 | 0.12 | -0.08 | 0.09 | -0.06 |
| PC(36:3) & PE(39:3) | 0.10 | 0.08 | 0.05 | -0.06 | 0.09 | -0.09 | 0.07 | 0.00 | 0.06 | 0.01 | 0.07 | -0.04 |
| SM(42:2) | 0.05 | -0.39 | 0.16 | 0.05 | 0.15 | 0.22 | 0.15 | -0.13 | 0.18 | -0.07 | 0.12 | 0.04 |
| PE(33:0) & PC(30:0) | 0.04 | 0.05 | 0.01 | 0.01 | 0.00 | 0.01 | 0.00 | 0.00 | 0.01 | 0.00 | 0.00 | 0.00 |
| PE(41:4) & PC(38:4) | 0.04 | -0.03 | 0.04 | -0.03 | 0.03 | -0.03 | 0.05 | -0.01 | 0.05 | 0.10 | 0.00 | -0.04 |
| SM(42:3) | 0.04 | -0.06 | 0.04 | 0.00 | 0.04 | 0.02 | 0.05 | -0.02 | 0.05 | 0.00 | 0.03 | 0.01 |
| PC(36:4) | 0.03 | 0.00 | 0.00 | 0.04 | -0.01 | 0.01 | 0.03 | 0.02 | 0.04 | 0.21 | 0.01 | 0.03 |
| PC(38:3) & PE(41:3) | 0.02 | 0.00 | 0.01 | -0.02 | 0.03 | -0.02 | 0.01 | -0.01 | 0.01 | 0.00 | 0.02 | -0.01 |
| PC-O(34:1) & PC-P(34:0) | 0.02 | -0.01 | 0.02 | -0.01 | 0.02 | 0.00 | 0.02 | -0.02 | 0.03 | -0.01 | 0.02 | -0.01 |
| PC-O(36:5) & PC-P(36:4) | 0.02 | 0.01 | 0.02 | 0.00 | 0.00 | 0.00 | 0.01 | 0.01 | 0.01 | 0.02 | 0.00 | -0.02 |
| PC(32:1) & PE(35:1) | 0.02 | 0.00 | 0.01 | -0.01 | 0.01 | 0.00 | 0.01 | -0.01 | 0.02 | 0.00 | 0.02 | 0.00 |
| PC(34:3) & PE(37:3) | 0.02 | 0.01 | 0.01 | 0.00 | 0.01 | 0.00 | 0.01 | 0.00 | 0.01 | -0.01 | 0.02 | 0.00 |
| SM(36:2) | 0.01 | -0.01 | 0.01 | 0.00 | 0.01 | 0.00 | 0.01 | 0.00 | 0.01 | 0.01 | 0.01 | 0.01 |
| SM(34:2) | 0.01 | 0.00 | 0.00 | 0.01 | 0.00 | 0.01 | 0.01 | 0.01 | 0.01 | 0.02 | 0.01 | 0.02 |
| SM(32:1) | 0.01 | -0.04 | 0.01 | 0.04 | 0.00 | 0.06 | 0.01 | 0.00 | 0.02 | 0.02 | 0.01 | 0.02 |
| PC(35:2) | 0.01 | 0.00 | 0.00 | 0.00 | 0.01 | 0.00 | 0.00 | -0.01 | 0.00 | 0.00 | 0.00 | 0.00 |
| PC(38:6) | 0.01 | 0.01 | 0.01 | 0.00 | 0.01 | -0.03 | -0.01 | 0.00 | 0.01 | 0.06 | 0.01 | -0.01 |
| PC(33:2) & PE(36:2) | 0.01 | -0.01 | 0.01 | 0.00 | 0.01 | 0.01 | 0.01 | -0.01 | 0.01 | 0.00 | 0.01 | 0.00 |
| PE(43:6) & PC(40:6) | 0.00 | 0.00 | 0.01 | -0.01 | 0.01 | -0.01 | 0.00 | -0.01 | 0.00 | 0.01 | 0.00 | -0.02 |
| PE(35:0) & PC(32:0) | 0.00 | -0.13 | 0.05 | 0.05 | 0.05 | 0.13 | 0.04 | -0.07 | 0.05 | 0.03 | 0.01 | 0.01 |
| PC-P(34:1) & PC-O(34:2) | 0.00 | 0.00 | 0.00 | 0.00 | 0.00 | 0.00 | 0.00 | 0.00 | 0.01 | 0.00 | 0.00 | 0.01 |
| PC-P(36:3) & PC-O(36:4) | 0.00 | 0.00 | 0.00 | 0.00 | 0.00 | 0.00 | 0.00 | 0.00 | 0.01 | 0.01 | 0.00 | 0.01 |
| PC-P(38:3) & PC-O(38:4) | 0.00 | 0.00 | 0.00 | 0.00 | 0.00 | 0.00 | 0.00 | 0.00 | 0.00 | 0.00 | 0.00 | 0.00 |
| PE(36:4) | 0.00 | -0.01 | 0.00 | 0.01 | 0.00 | 0.01 | 0.00 | -0.01 | 0.00 | 0.01 | 0.00 | 0.00 |
| PE(36:3) & PC(33:3) | 0.00 | -0.01 | 0.00 | 0.00 | 0.00 | 0.01 | 0.00 | -0.01 | 0.00 | 0.00 | 0.00 | 0.00 |
| PC(40:5) & PE(43:5) | 0.00 | 0.00 | 0.00 | 0.00 | 0.00 | 0.00 | 0.00 | 0.00 | 0.00 | 0.00 | 0.00 | 0.00 |
| PE(38:3) & PC(35:3) | 0.00 | 0.00 | 0.00 | 0.00 | 0.00 | 0.00 | 0.00 | 0.00 | 0.00 | 0.00 | 0.00 | 0.00 |
| PE(43:4) | 0.00 | 0.00 | 0.00 | 0.00 | 0.00 | 0.00 | 0.00 | 0.00 | 0.00 | 0.00 | 0.00 | 0.00 |
| SM(45:0) | 0.00 | 0.00 | 0.00 | 0.00 | 0.00 | 0.00 | 0.00 | 0.00 | 0.00 | 0.00 | 0.00 | 0.00 |
| PC-P(20:0) | 0.00 | 0.00 | 0.00 | 0.00 | 0.00 | 0.00 | 0.00 | 0.00 | 0.00 | 0.00 | 0.00 | 0.00 |
| PE(40:3) & PC(37:3) | 0.00 | 0.00 | 0.00 | 0.00 | 0.00 | 0.00 | 0.00 | 0.00 | 0.00 | 0.00 | 0.00 | 0.00 |
| PC-O(36:3) & PC-P(36:2) | 0.00 | 0.00 | 0.00 | 0.00 | 0.00 | 0.00 | 0.00 | 0.00 | 0.00 | 0.00 | 0.00 | 0.00 |
| PC-O(40:5) | 0.00 | 0.00 | 0.00 | 0.00 | 0.00 | 0.00 | 0.00 | 0.00 | 0.00 | 0.00 | 0.00 | 0.00 |
| SM(39:2) | 0.00 | 0.00 | 0.00 | 0.00 | 0.00 | 0.00 | 0.00 | 0.00 | 0.00 | 0.00 | 0.00 | 0.00 |
| PC(37:4) | 0.00 | 0.00 | 0.00 | 0.00 | 0.00 | 0.00 | 0.00 | 0.00 | 0.00 | 0.00 | 0.00 | 0.00 |
| PC-O(34:3) & PC-P(34:2) | 0.00 | 0.00 | 0.00 | 0.00 | 0.00 | 0.00 | 0.00 | 0.00 | 0.00 | 0.00 | 0.00 | 0.00 |
| SM(41:3) | 0.00 | 0.00 | 0.00 | 0.00 | 0.00 | 0.00 | 0.00 | 0.00 | 0.00 | 0.00 | 0.00 | 0.00 |
| SM(40:3) | 0.00 | 0.00 | 0.00 | 0.00 | 0.00 | 0.01 | 0.00 | 0.00 | 0.00 | 0.00 | 0.00 | 0.00 |
| SM(34:1) | 0.00 | -0.62 | 0.09 | 0.26 | 0.05 | 0.34 | 0.14 | -0.06 | 0.12 | 0.17 | 0.04 | 0.16 |
| SM(45:1) | 0.00 | 0.00 | 0.00 | 0.00 | 0.00 | 0.00 | 0.00 | 0.00 | 0.00 | 0.00 | 0.00 | 0.00 |
| PE(40:5) & PC(37:5) | 0.00 | 0.00 | 0.00 | 0.00 | 0.00 | 0.00 | 0.00 | 0.00 | 0.00 | 0.00 | 0.00 | 0.00 |
| SM(33:1) | 0.00 | -0.02 | 0.00 | 0.00 | 0.00 | 0.00 | 0.00 | 0.00 | 0.00 | 0.00 | 0.00 | 0.01 |
| SM(43:4) | 0.00 | 0.00 | 0.00 | 0.00 | 0.00 | 0.01 | 0.00 | 0.00 | 0.00 | 0.00 | 0.00 | 0.00 |
| PE(37:4) & PC(34:4) | 0.00 | 0.00 | 0.00 | 0.00 | 0.00 | 0.00 | 0.00 | 0.00 | 0.00 | 0.00 | 0.00 | 0.00 |
| SM(37:1) | 0.00 | 0.00 | 0.00 | 0.00 | 0.00 | 0.01 | 0.00 | 0.00 | 0.00 | 0.00 | 0.00 | 0.01 |
| SM(42:5) | -0.01 | 0.00 | -0.01 | 0.01 | -0.01 | 0.01 | -0.01 | 0.00 | -0.01 | 0.01 | 0.00 | 0.01 |
| SM(446 | -0.01 | -0.01 | -0.01 | 0.01 | -0.01 | 0.01 | -0.01 | 0.00 | -0.01 | 0.01 | 0.00 | 0.01 |
| SM(39:1) | -0.01 | -0.01 | -0.01 | 0.01 | -0.01 | 0.02 | -0.01 | 0.00 | -0.01 | 0.01 | -0.01 | 0.02 |
| SM(42:4) | -0.01 | -0.01 | -0.02 | 0.02 | -0.01 | 0.02 | -0.01 | 0.00 | -0.01 | 0.01 | -0.01 | 0.02 |
| SM(444 | -0.01 | -0.01 | -0.01 | 0.02 | -0.01 | 0.03 | -0.01 | 0.00 | -0.01 | 0.02 | -0.01 | 0.02 |
| SM(41:1) | -0.01 | -0.02 | -0.01 | 0.01 | -0.02 | 0.02 | -0.01 | 0.00 | -0.01 | 0.01 | -0.01 | 0.02 |
| PC(38:5) & PE(41:5) | -0.02 | 0.01 | -0.05 | 0.03 | -0.03 | 0.03 | -0.02 | 0.02 | -0.04 | 0.07 | -0.02 | 0.04 |
| SM(38:1) | -0.02 | -0.04 | -0.02 | 0.04 | -0.03 | 0.04 | -0.01 | 0.00 | -0.01 | 0.02 | -0.02 | 0.04 |
| SM(445 | -0.02 | -0.05 | -0.03 | 0.04 | -0.02 | 0.05 | -0.02 | 0.00 | -0.03 | 0.03 | -0.01 | 0.04 |
| SM(36:1) | -0.03 | -0.11 | 0.00 | 0.06 | 0.00 | 0.05 | 0.01 | -0.01 | 0.00 | 0.04 | 0.00 | 0.03 |
| SM(42:1) | -0.03 | -0.10 | -0.01 | 0.11 | -0.03 | 0.17 | 0.00 | -0.02 | 0.00 | 0.10 | -0.03 | 0.07 |
| SM(40:1) | -0.03 | -0.08 | -0.02 | 0.07 | -0.03 | 0.10 | 0.00 | -0.02 | -0.01 | 0.05 | -0.03 | 0.06 |
| PC(36:5) & PE(39:5) | -0.04 | 0.02 | -0.09 | 0.07 | -0.07 | 0.07 | -0.04 | 0.03 | -0.08 | 0.07 | -0.04 | 0.10 |

*Table S2. The full list of loading values for lipids in the PCAs shown in* Fig. S1*. These figures quantify the importance of variables within each test. The first two digits of each three number code refer to the number of carbons in the fatty acid residues of the respective species. The third digit is the number of double bonds. Where two species are quoted, these are isobaric. The appropriate feeding control group was used against both SGA and MHG samples. BF, breastfed; M, mixed intake; FF, formula-fed.*
